# Reduction in the incidence of myocardial infarction with sodium–glucose linked cotransporter-2 inhibitors: evident and plausible

**DOI:** 10.1186/s12933-019-0812-6

**Published:** 2019-01-11

**Authors:** Richard E. Gilbert, Kim A. Connelly

**Affiliations:** 1 Division of Endocrinology, St. Michael’s Hospital, 30 Bond Street, Toronto, ON M5B 1W8 Canada; 2 Division of Cardiology, St. Michael’s Hospital, 30 Bond Street, Toronto, ON M5B 1W8 Canada

**Keywords:** Sodium–glucose linked cotransporter-2, Myocardial infarction, Cardiovascular outcome trials, Stroke

Coincident with the recent reporting of the Dapagliflozin Effect on Cardiovascular Events (DECLARE) trial in the *New England Journal of Medicine* [1], the *Lancet* published a systematic review and meta-analysis of cardiovascular outcome trials for the three widely marketed SGLT2 inhibitors: canagliflozin, empagliflozin and dapagliflozin [2].

While able to reduce hospitalization for heart failure, kidney disease progression and cardiovascular death, sodium–glucose linked cotransporter-2 (SGLT2) inhibitors are not generally regarded as agents that reduce the atherosclerotic components of MACE: myocardial infarction and stroke. The meta-analysis of the SGLT2 inhibitor cardiovascular outcome trials suggests, however, that for this drug class myocardial infarction and stroke should be viewed separately [2]. Not only was the reduction in myocardial infarction statistically significant 0.89 (95% confidence intervals: 0.80, 0.98) but the point estimates for all three trials also lay on the favourable side of unity. These findings contrast those for stroke and amputation where the hazard ratios were non-significant and where heterogeneity in the direction of effect was also evident (Fig. 1).

**Fig. 1:**
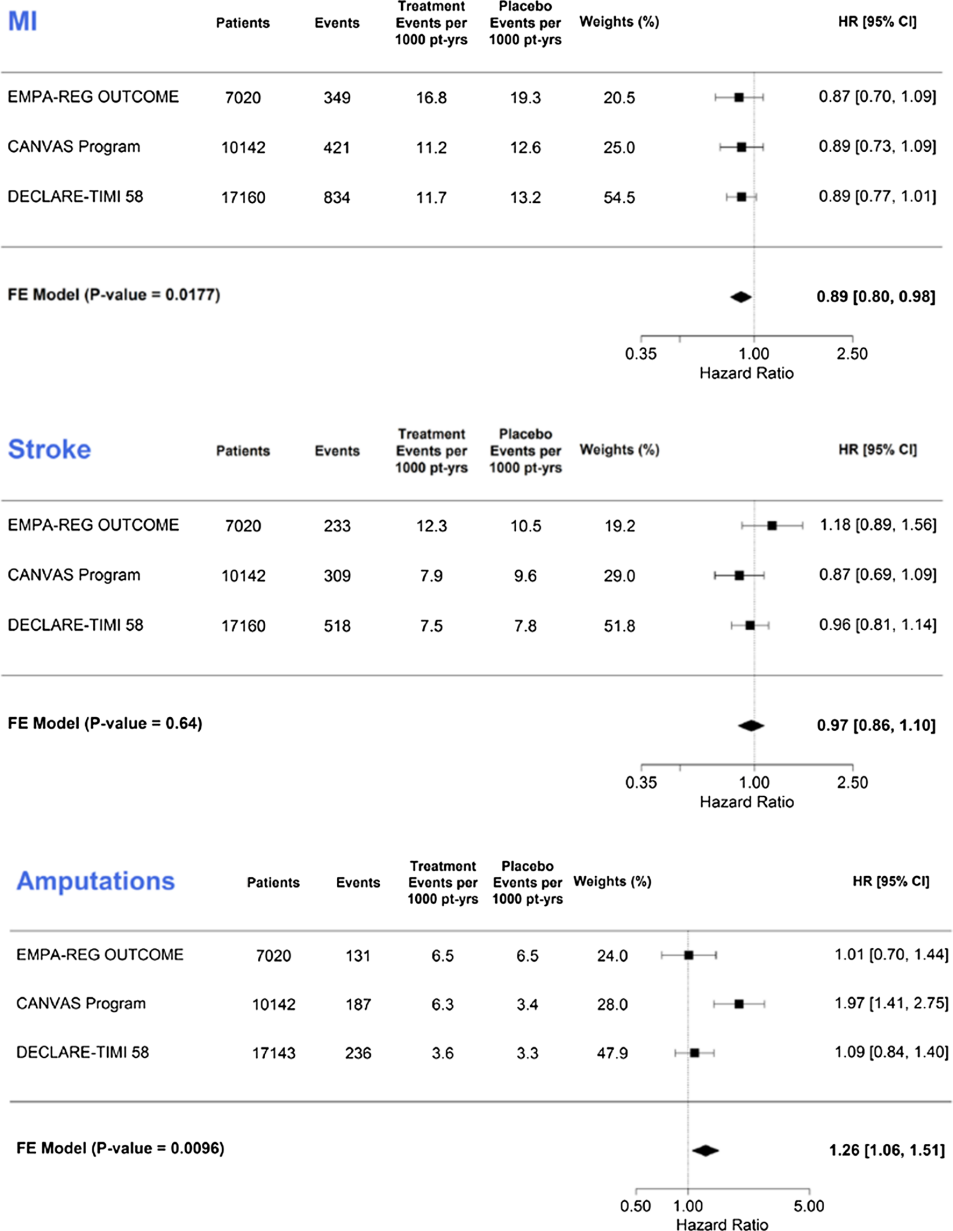
Myocardial infarction, stroke and amputation events in EMPA-REG Outcome, CANVAS and DECLARE studies, reproduced with permission from [2]

The observed difference in hazard ratios among myocardial infarction, stroke and amputation suggest that a primary anti-atherosclerotic effect of the SGLT2 inhibitors is unlikely since such an effect would have been expected to reduce myocardial infarction and stroke similarly, as is the case with cholesterol lowering [3] and antihypertensive therapy [4]. And though it is possible that the reduction in myocardial infarction is a chance finding, the adjudication of events, the robust numbers and the statistical testing all suggest that this is not the case. Accordingly, these data from randomized controlled trials with the support of similar findings in the so-called real world setting [5] should be regarded as hypothesis-generating.

Infarction occurs when the demands of the myocardium exceed the supply of O_2_ needed to maintain viability. As such, its likelihood can be reduced by either augmenting O_2_ supply or reducing its demand. Nitrates, for instance, are thought to improve symptoms in patients with flow-limiting coronary artery disease primarily by reducing preload that, in turn, leads to a diminution in left ventricular volume, wall tension and O_2_ demand [6]. Nicorandil, for instance, a nitrate derivative with venodilating properties, reduces preload and the risk of myocardial infarction following percutaneous coronary intervention [7]. Through the promotion of an osmotic diuresis, SGLT2 inhibitors also reduce preload and while detailed human studies are in progress, animal studies have demonstrated the ability of this class of agent to similarly reduce left ventricular volumes in systole and diastole and thereby wall tension [8]. Accordingly, we hypothesize that the diminution in myocardial infarction with SGLT2 inhibitors is a consequence of preload reduction in patients with established cardiovascular disease. This drug class would therefore not be expected influence the risk of stroke or critical limb ischemia or be particularly effective in patients with multiple risk factors alone.

In conclusion, we view the meta-analysis-based finding of a statistically significant reduction in myocardial infarction risk in diabetic individuals treated with SGLT2 inhibitors as real, and consistent with the known effects of this drug class on cardiac preload.

## Abbreviations

SGLT2: sodium–glucose linked cotransporter-2
CANVAS: Canagliflozin Cardiovascular Assessment Study
DECLARE: Dapagliflozin Effect on Cardiovascular Events
MACE: major adverse cardiovascular events
